# Kinetic Study of Glucosamine Production Using *Aspergillus sydowii* BCRC 31742 under Solid-State Fermentation

**DOI:** 10.3390/molecules25204832

**Published:** 2020-10-20

**Authors:** Jia Wei Peng, Ho Shing Wu

**Affiliations:** Department of Chemical Engineering and Materials Science, Yuan Ze University, 135 Yuan Tung Road Chung Li, Taoyuan 32003, Taiwan; s1055225@mail.yzu.edu.tw

**Keywords:** glucosamine, *Aspergillus sydowii*, solid-state fermentation, moisture

## Abstract

In the present study, we aimed to obtain a high yield and productivity for glucosamine using a low-cost solid-state culture with *Aspergillus sydowii* BCRC 31742. The fermentation conditions, such as inoculum biomass, moisture content, and supplemental volume and mineral salt, were chosen to achieve high productivity of glucosamine (GlcN). When the initial supplemental volume used was 3 mL/g substrate, the yield and productivity of GlcN were 48.7 mg/gds and 0.69 mg/gds·h, respectively. This result will be helpful for the industrialization of the process.

## 1. Introduction

Glucosamine (GlcN), also known as aminosaccharide, is an essential functional monosaccharide. Glucosamine and its derivatives have a vast range of applications, especially in medicine, industry, and other fields [1]. For decades, most GlcN preparations have been derived from the exoskeletons of shellfish or other marine resources [2]. However, as the demand for GlcN continues to increase, GlcN production using this extraction method may be limited by the availability of variable feedstock. Besides this, GlcN from shellfish may not be suitable for people with shellfish allergies or may contain environmental contaminants (heavy metals), and the harvesting period of shellfish is seasonal [3,4,5].

GlcN can be present as chitosan and chitosan monomers in the cell walls of fungi. Such fungi include subdivisions of Ascomycota (e.g., *Aspergillus* sp. [6,7] *Aspergillus terreus* [8]) and chimeric gates (e.g., *Rhizopus* species, *Mucor* species, and *Syringa* species). More recently, many studies have been conducted to study GlcN production using fungal fermentation to overcome the limitations resulting from the hydrolysis of chitin. The following three types of wild-type fungi are used for GlcN production: *Rhizopus oligosorus* [9], *Monascus* [10], and *Aspergillus sydowii* [11]. Hsieh et al. [6] first published the production of GlcN using *A. sydowii* BCRC 31742. Chang et al. [12] used *A. sydowii* BCRC 31742 in submerged fermentation to obtain a GlcN concentration of 5.48 g/L and a biomass concentration of 21.6 g/L. Lately, the pellet size and rheology of *A. sydowii* during fungal fermentation have been studied [13]. 

Two fermentation techniques have been developed: submerged fermentation and solid-state fermentation. The main difference between the two types of fermentation is the amount of water in the fermentation medium. However, using submerged fermentation in mass culture has several limitations, such as oxygen solubility, wastewater treatment, and greater energy consumption in the ventilation of equipment and machines [12]. Therefore, the cost is relatively higher. On the other hand, solid-state fermentation not only produces GlcN directly but, also, consumes agricultural waste. Solid-state fermentation is defined as any fermentation process performed on a nonsoluble material that acts both as physical support and as source of nutrients in the absence of free liquid and is already applied in production in many food industries [14]. The use of this method to produce GlcN can reduce the cost and have a beneficial impact on the environment. Our previous work published a patent (US 20200087693 A1) for GlcN production with wheat bran using *A. sydowii* BCRC 31742 [15], which presented such conditions as the amount of molasses, cultivation time, kind of fungus, medium thickness of wheat bran, and supplemental addition. This work aims to discuss the initial supplemental volume, moisture content, supplemental mineral salts, and fungal morphology in solid-state fermentation.

## 2. Results and Discussion

The compositions of the molasses and wheat bran were analyzed in our previous work [15]. From previous reports, compared to molasses, wheat bran has more macromolecules, such as crude protein (17.1 wt%), crude fat (58 wt%), and saturated fat (0.87 wt%). It can be inferred that the wheat bran needs cooking or sterilization using a high temperature and pressure to decompose into small molecules so that fungi can easily take. Besides this, sugar in wheat bran (2.95 wt%) is quite lacking, so when using wheat bran in solid-state fermentation, the addition of some sugar like molasses (39.1 wt%) has an absolutely positive relationship with an increase of biomass. The mineral salts that exist in the wheat bran (Na: 9.8 × 10^−3^ wt%, K, and Mg: 0 wt%) are also present in lower quantities than in molasses (Na: 0.07%, K: 25.9 wt%, and Mg: 3.8 wt%), so supplementing a metal-supplemental solution is necessary for the growth of fungi. 

### 2.1. Effect of Particle Size of Wheat Bran

**Figure 1:**
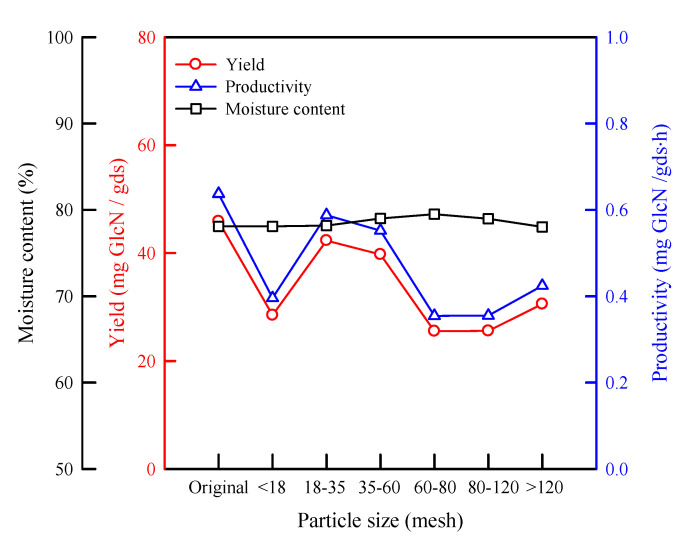
Effect of the particle size of wheat bran on the yield and productivity of glucosamine (GlcN). *Aspergillus sydowii*; 30 °C; wheat bran 5 g; 72 h; initial supplemental volume (pH 4.2, KH_2_PO_4_ 0.2 wt%, NaCl 0.1 wt%, and MgSO_4_·7H_2_O 0.1 wt%) 3 mL/g; substrate; and inoculum biomass 18 mg/g substrate.

Typically, smaller particles provide a greater substrate surface area for microbial growth. However, if the particle size of the substrate is too small, the substrate tends to aggregate, which will result in the weak growth of fungi due to hypoxia. Therefore, a compromise on granularity should be made [14]. Figure 1 shows the effect of the particle size of wheat bran on the yield and productivity of GlcN. The productivities of GlcN for the particle sizes of the original sample, including all particle sizes, 18–35 mesh, and 35–60 mesh, are larger than those for the others. Since using the same irregular particles for every run is a challenge, the particle size of 18–35 mesh was chosen for further study in this experiment.

### 2.2. Effect of Preculture Time of Liquid Fermentation

**Figure 2:**
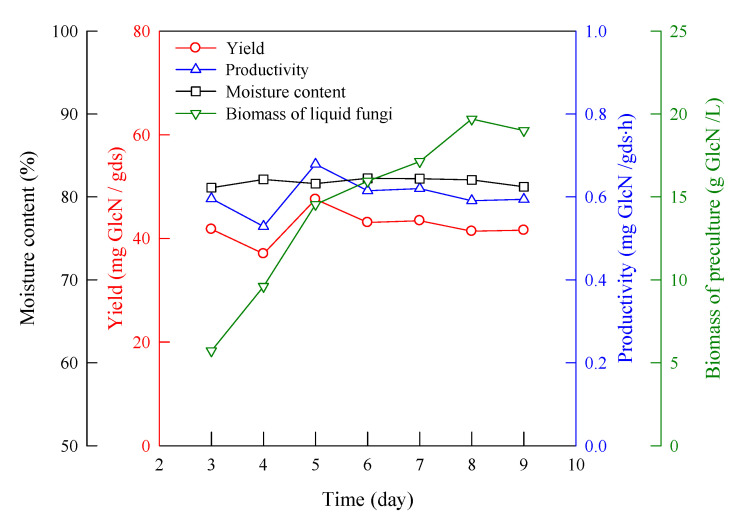
Effect of preculture time of liquid fermentation on the yield and productivity of GlcN. *A. sydowii*, 30 °C, wheat bran (18–35 mesh) 5 g, 72 h, initial supplemental volume 3 mL/g substrate, pH 4.2, KH_2_PO_4_ 0.2 wt%, NaCl 0.1 wt%, MgSO_4_·7H_2_O 0.1 wt%, and inoculum biomass 18 mg/g substrate.

Figure 2 shows the effect of the preculture submerged-fermentation time on the yield and productivity of GlcN and the moisture content. The preculture time that obtained the maximal GlcN concentration was eight days, but that which obtained the maximal yield of GlcN was five days in solid-state fermentation, not eight days. The yield and productivity of GlcN were 47.5 mg/gds (gram dry substrate) and 0.67 mg/gds·h, respectively. Although the GlcN concentration of the liquid fungi increased with the increasing time (green line), it did not have a positive impact on solid-state fermentation (red line). The specific growth rate of the biomass in submerged fermentation was 0.0228 h^−1^. After five days, the growth rate became slow. After eight days, the phase was stationary. Hence, the inoculum time chosen was five days in this work. 

### 2.3. Effect of Inoculum Biomass

**Figure 3:**
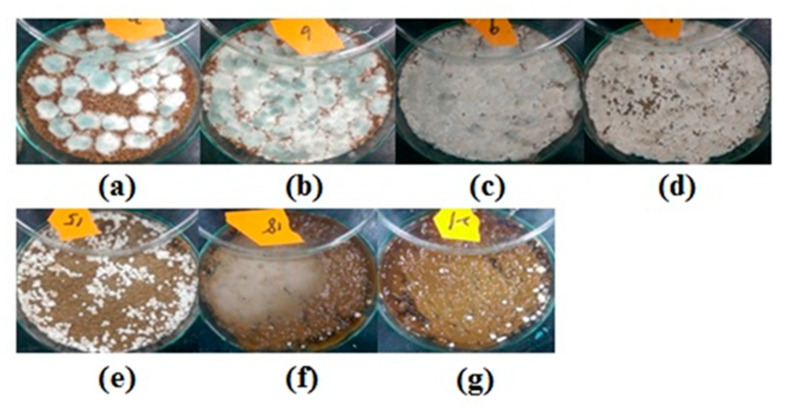
Photographs of the effect of inoculum biomass. The concentrations of inoculum biomass (milligrams of biomass per gram of substrate) are (**a**) 9, (**b**) 18, (**c**) 27, (**d**) 36, (**e**) 45, (**f**) 54, and (**g**) 63; fermentation time: 72 h.

Increasing the amount of fungi in the inoculum causes rapid proliferation. However, after a threshold over which competition for nutrients occurs, the metabolic activity of the organism declines. As a consequence, finding the appropriate inoculum biomass is essential to increasing the biomass. From Figure 3, if the inoculum biomass is controlled at less than 18 mg/g substrate, the substrate is too much for fungi growth. It is reasonable that utilization gets worse. In contrast, when the inoculum biomass is larger than 18-mg/g substrate, the culture medium becomes increasingly viscous due to the increasing inoculum biomass. Thus, fungi get nutrients from wheat bran or gets oxygen from the atmosphere with difficulty. The productivity and yield of GlcN decreased with the increasing inoculum biomass. The best inoculum biomass was 18 mg/g substrate, for which the yield and productivity of GlcN were 45.9 mg/gds and 0.67 mg/gds·h, respectively.

### 2.4. Effect of Initial Supplemental Solution Volume

The moisture content (MC) and water activity of the wheat bran medium corresponding to each level of water supplemented to the medium were determined using the methods. The water activity was larger than 0.99 when the MC was larger than 59% (wet basis), as shown in Table 1. The relationship between the MC (wet basis) and water activity of the wheat bran at 25 °C is expressed as MC = −0.0689 − 0.4203 log(1 − a_w_) based on the calculation in a previous work [16].

**Figure 4:**
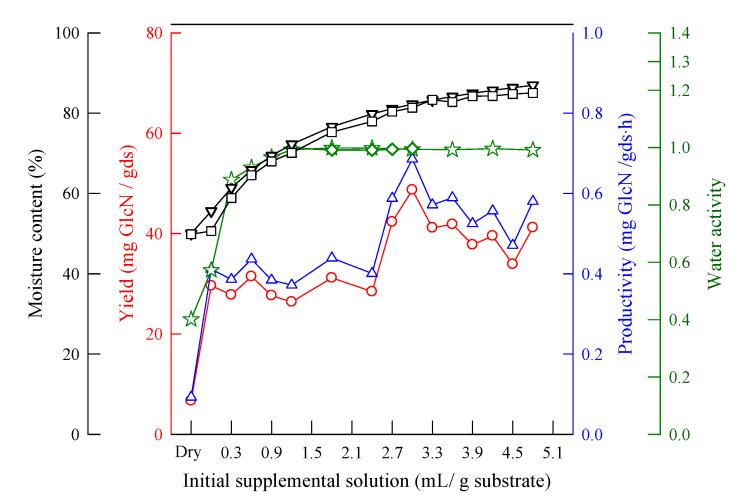
The effect of the initial supplemental volume on the yield and productivity of GlcN. pH 4.2, KH_2_PO_4_ 0.2 wt%, NaCl 0.1 wt%, MgSO_4_·7H_2_O 0.1 wt%, *A. sydowii*, 30 °C, (18–35 mesh) wheat bran 5 g, 72 h, and inoculum biomass 18 mg/g substrate. (**O**) yield; (**Δ**) productivity; (**☆**) water activity of wheat bran and water (before fermentation); (**◇**) water activity of wheat bran, water, and fungi (after fermentation); (**∇**) moisture content of wheat bran and water (before fermentation); and (**□**) moisture content of wheat bran, water, and fungi (after fermentation).

**Figure 5:**
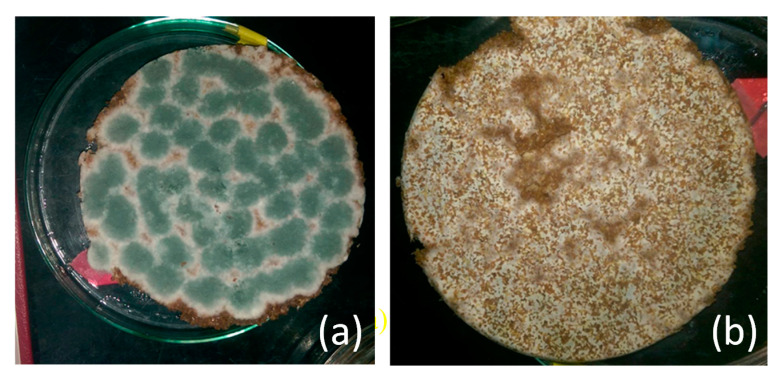
Morphology of (**a**) the front side and (**b**) the reverse side of wheat bran after 5 days of solid-state fermentation.

Figure 4 shows that, when the initial supplemental volume used was 3 mL/g substrate, the yield and productivity of GlcN were 48.7 mg/gds and 0.69 mg/gds·h, respectively. The water activity and MC were larger than 0.99 and 80%, respectively, during fermentation. A larger volume of supplement solution added is better than a lower one. Based on the fermentation surface area, the GlcN yield and productivity of GlcN were 38 kg/m^2^ and 0.54/m^2^·h, respectively. The front side and reverse side of the wheat bran are shown in Figure 5, with a thickness of around 2.4 mm. The fungal growth proceeded well.

**Figure 6:**
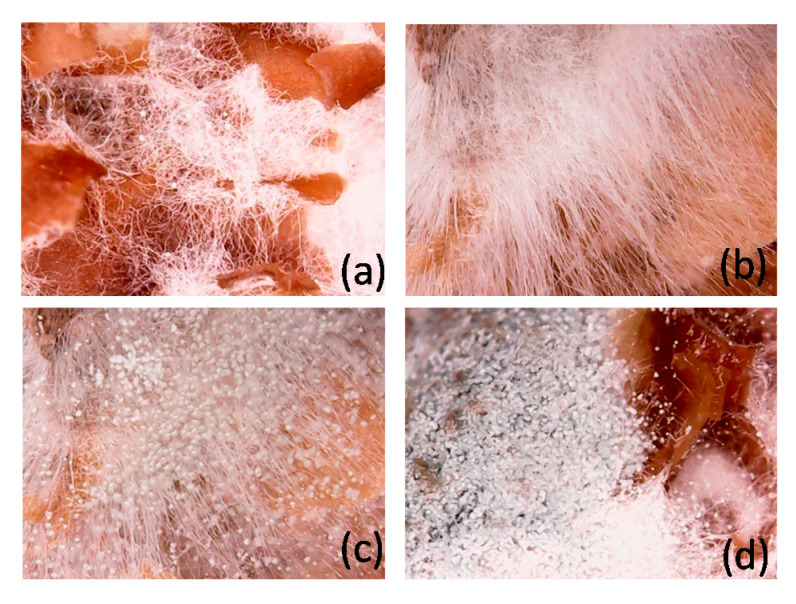
Morphology of fungal growth under a microscope: (**a**) 2 days (100×), (**b**) 2 days (500×), (**c**) 4 days (500×), and (**d**) 8 days (500×).

The morphology of the fungal growth observed under a microscope (500×) indicated that fungi began to produce spore capsules at two days of cultivation, shown in Figure 6, which approached the highest glucosamine productivities. After three days, the color of the spore capsules changed to grey. The fungi gradually died over a long time, shown in Figure 6d. In this present work, the biomass content in solid-state fermentation was estimated based on that in submerged fermentation [17]. The GlcN content in mycelial weight in submerged fermentation was around 150 mg/g. The fungi grown in solid-state fermentation was calculated and equaled 0.32 g/gds (= 48.7/150).

### 2.5. Effect of Volume Ratio of Inoculum to Initial Supplemental Solution

**Figure 7:**
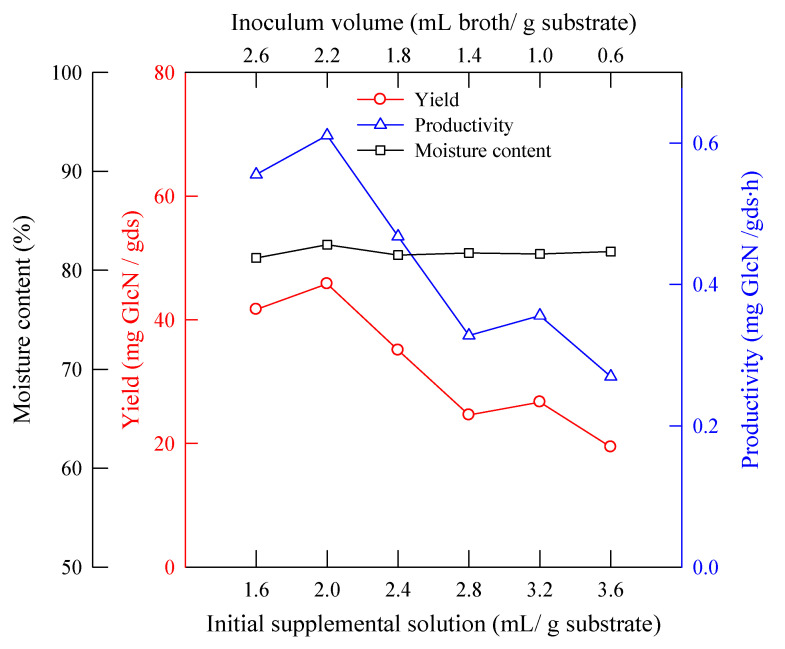
The effect of the volume ratio of inoculum to initial supplemental solution on the yield and productivity of GlcN. *A. sydowii*, 30 °C, wheat bran (18–35 mesh) 5 g, 72 h, initial supplemental volume 3 mL/g substrate, pH 4.2, KH_2_PO_4_ 0.2 wt%, NaCl 0.1 wt%, and MgSO_4_·7H_2_O 0.1 wt%.

Besides the amount of water, the ratio of water to substrate is an indispensable factor in solid-state fermentation. There are two major liquid phases in solid-state fermentation: the inoculum and the supplemental solution. The total liquid volume of inoculum and supplemental solution was limited to 4.2 mL/g substrate. The inoculum concentration was 15 ± 2 mg/mL. Then, the ratio of inoculum and supplemental solution volume was changed to obtain the appropriate proportion. Figure 7 shows the effect of the volume ratio of the inoculum to the initial supplemental solution on the yield and productivity of GlcN and the moisture content. The best proportion of liquid was an inoculum volume of 2.2 mL/g substrate to 2 mL/g substrate. 

### 2.6. Effect of Mineral Salts in Supplemental Solution

**Figure 8:**
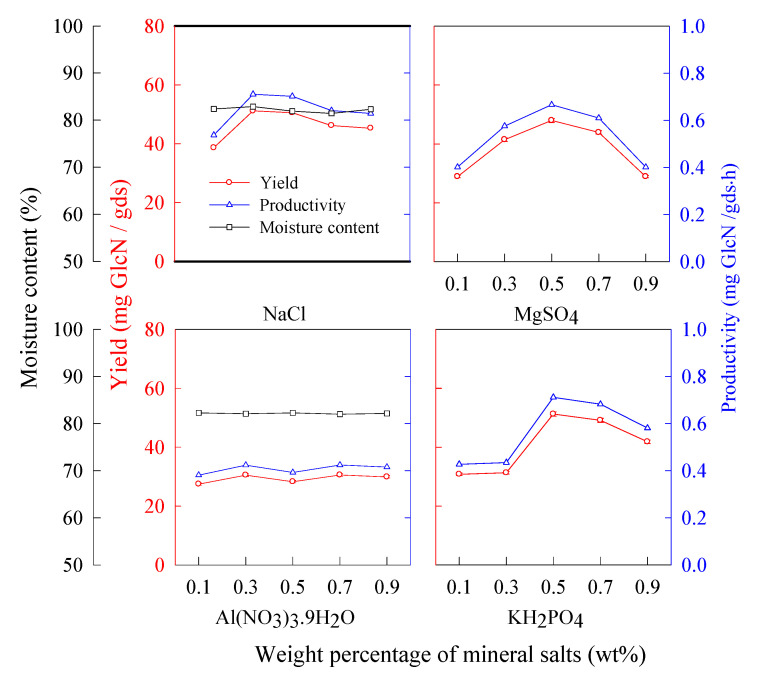
The effect of mineral salts in the supplemental solution on the yield and productivity of GlcN. *A. sydowii*, 30 °C, wheat bran (18–35 mesh) 5 g, 72 h, initial supplemental volume 3 mL/g substrate, pH 4.2, and inoculum biomass 18 mg/g substrate.

Microbial transformation of metals and minerals is an essential part of the natural biosphere process. Salts can allow the fungi to accumulate increased GlcN in their cell walls [12,18]. According to the results in Figure 8, the GlcN yield using NaCl, KH_2_PO_4_, and MgSO_4_ was larger than that using Al(NO_3_)_3_.9H_2_O. The GlcN yield reached 51.2 and 51.1 mg/gds using KH_2_PO_4_ (0.5 wt%) and NaCl (0.3 wt%), respectively. 

## 3. Materials and Methods

### 3.1. Materials

*A. sydowii* BCRC 31742 was purchased from the Bioresource Collection and Research Center (BCRC) in Hsinchu, Taiwan. The GlcN standard (D-(+)-GlcN hydrochloride, 99% in purity), 1-naphthyl isothiocyanate (98% in purity), and 3,5-dinitrobenzonitrile (97% in purity) were purchased from Sigma (ST. Louis, MO., USA). HPLC-grade reagents pyridine (99.5% in purity) and acetonitrile (99.8% in purity) were purchased from Riedel-de Haen (Seelze, Germany) and Mallinckrodt Chemicals (Bedminster, NJ, USA), respectively. The compound medium contained molasses (Light Green, Taoyuan, Taiwan), soybean hydrolysate (L. Seatex Co., Ltd., Taipei, Taiwan), NaCl (Mallinckrodt, Bedminster, NJ, USA), KH_2_PO_4_ (Riedel-de Haen, Seelze, Germany), and MgSO4·7H2O (R.D.H, Germany). Wheat bran was purchased from Ti Yi Industrial (Taoyuan, Taiwan).

### 3.2. Preculture of Fungi

The aim of fungus preculturing is to produce the inoculum that is transferred to the solid-state fermentation medium. The preculture medium contained molasses (150 mL/L), soybean hydrolysate (5 mL/L), MgSO_4_ (0.1 g/L), Al(NO_3_)_3_ (0.1 g/L), and methanol (1 mL/L), with pH adjusted to 7 using NaOH. Typically, 150 mL of preculture medium was put into a 250 mL Erlenmeyer flask. The medium in the flask was sterilized using an autoclave at 121 °C and 2 atm for 20 min. After sterilization, 1 Eppendorf of 1 mL (or 10–15 colonies of *A. sydowii* BCRC 31742) was taken and mixed with the preculture medium in a flask. The incubation was carried out at 30 °C and 200 rpm [19]. The sample was withdrawn at a selected time to determine the GlcN concentration.

### 3.3. Solid-State Fermentation

Five grams of the wheat bran substrate were placed in a petri dish (Ø 9 cm), and then, the supplemental solution (3 mL/g substrate, 0.2 wt% KH_2_PO_4_, 0.1 wt% NaCl, and 0.1 wt% MgSO_4_·7H_2_O) was added. Then, the plate was sterilized. The fungus (1 mL/g substrate), which was withdrawn from the preculture solution, was inoculated onto the solid substrate in a laminar flow hood. The inoculation concentration of the fungus was 15 ± 2 mg/mL. After inoculation, the plates were placed in an incubator at 30 °C for a selected time. The morphology of the fungal growth was monitored online by means of a digital microscope (Met-MS500, Seatools, Seagate Technology, Cupertino, CA, USA).

**Figure 9:**
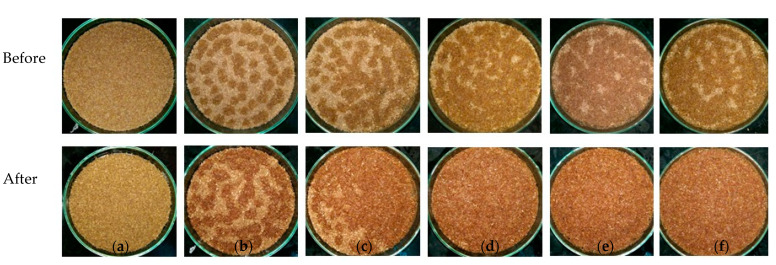
The moisture appearance of wheat bran surfaces in petri dishes (Ø 9 cm) before and after sterilization: (**a**) 0, (**b**) 0.9, (**c**) 1.8, (**d**) 2.7, (**e**) 3.6, and (**f**) 4.5 mL water/g substrate.

Figure 9 shows that the amount of supplemental solution volume should be larger than 2.7 mL/(g wheat bran), so that the supplemental solution can be fully distributed on the surface of the wheat bran after sterilization. Otherwise, some areas of the wheat bran do not have sufficient contact with the supplemental solution. The nutrition in the wheat bran cannot then be released in fermentation, and the fungi grow very poorly.

### 3.4. Determination of Fungal GlcN

The determination of fungal GlcN was carried out by means of HPLC, as reported in our previous studies [6,20], wherein the hydrochlorination process was used by a conventional thermal method. The analytical HPLC column was a LiChrospher^®^ 100 RP-18 column (5 μm, 4 mm (internal diameter) × 250 mm). The detector used was a UV-vis detector SPD-10 A (Shimadzu, Japan), as performed at a wavelength (λ) of 230 nm. The mobile phase was water and acetonitrile (87:13 *v*/*v*), with a flow rate of 1.3 mL/min. The column temperature was maintained at 40 °C. 

### 3.5. Determination of Water Activity Using the Diffusion Method

Water plays a crucial role in biological systems [21]. Many mechanisms of action and interaction of these fungi with organic molecules cannot work without water. Therefore, water is essential to fungal metabolism in solid-state fermentation. Insufficient water causes a poor diffusion of solutes and gases, slows cell metabolism, and can even stop growth. However, excessive water causes the porosity to be decreased and inhibits oxygen transfer between the substrate and the atmosphere. In general, the type of microorganism that can be grown in a solid-state fermentation system is determined by the water activity a_w_. The water activity is defined as the relative humidity of the gaseous atmosphere in equilibrium with the substrate. The a_w_ of the substrate quantitatively expresses the water requirement for microbial activity [22]:

(1) $$a_{w}=\frac{\;-\;Vm\emptyset }{55.5}\;$$

where V is the number of ions formed, *m* is the molar concentration of the solute, Ø is the molar osmotic coefficient, and the molar concentration of water is 55.5 mol. Pure water has an a_w_ of 1.00, and the a_w_ value decreases with the addition of the solutes. Bacteria mainly grow at higher a_w_ values, while filamentous fungi and some yeasts can grow at lower a_w_ values (0.6~0.7). The water activity of the substrate has been proposed as the condition determining the growth and viability of microorganisms [23].

The samples were diffused and equilibrated with different standard salt-saturated solutions in a closed and isothermal Conway dish [24]. According to the variation of the samples’ weight during equilibration in an incubator, the water activity and the standard salt-saturated solution were plotted as the ordinate and abscissa, respectively. Then, the water activity (a_w_) of the sample was calculated. This method is suitable for samples with high water activity (a_w_ > 0.5). One gram of sample and a known weight of aluminum foil were put in the inner well of the Conway dish, and 3 mL of standard salt-saturated solution was placed in the outer wall. Before equilibrating, parafilm was used to close the Conway dish. Afterward, the samples were equilibrated at 25 °C in an incubator for 2 h.

### 3.6. Moisture Content

The sample (1 g) was dried in an oven at 60 °C. The amount of sample was weighed at 3-h intervals until the result was obtained with accuracy within 5%. The moisture content was calculated by dividing the weight of the water in a mixture of biomass and substrate by the weight of the total mass (substrate and biomass). 

### 3.7. Determination of Particle Size of Wheat Bran

The particle size distribution of the wheat bran was determined using a vibratory sieve separator machine with sieve plates of 18 mesh, 35 mesh, 60 mesh, 80 mesh, 120 mesh, and 200 mesh. The sample (200 g) was added to the sieve separator and then vibrated for 30 min. The particles were collected between two meshes. The proportions of the particle size of wheat bran were 31.5 wt% (<18 mesh), 42.1 wt% (18–35 mesh), 21.4 wt% (35–60 mesh), 3.4 wt% (60–80 mesh), 1.4 wt% (80–120 mesh), and 0.2 wt% (>120 mesh).

### 3.8. Calculation of Yield, Productivity, and Content of GlcN

The yield, productivity, content, and concentration of GlcN are the representative results in this study. The yield of GlcN was calculated by dividing the amount of GlcN obtained by the weight of the initial dry substrate. The productivity of GlcN was calculated by dividing the amount of obtained GlcN by the weight of initial dry substrate and the incubation time. The content of GlcN was calculated by dividing the amount of obtained GlcN by the weight of initial dry substrate and the biomass. The concentration of GlcN was calculated by dividing the amount of obtained GlcN by the volume of broth. Their equations are given as follows.

(2) $$\mathrm{Yield}\;\left(\mathrm{mg}/\mathrm{gds}\right)=\frac{\mathrm{weight}\;\mathrm{GlcN}}{\mathrm{weight}\;\mathrm{of}\;\mathrm{dry}\;\mathrm{substrate}}$$

(3) $$\mathrm{Productivity}\;\left(\mathrm{mg}/\mathrm{gds}.h\right)=\frac{\mathrm{weight}\;\mathrm{of}\;\mathrm{GlcN}}{\mathrm{dry}\;\mathrm{weight}\;\mathrm{of}\;\mathrm{substrate}\times \mathrm{incubation}\;\mathrm{time}}$$

(4) $$\mathrm{Content}\;(\%)\;=\frac{\mathrm{weight}\;\mathrm{of}\;\mathrm{GlcN}}{\mathrm{weight}\;\mathrm{of}\;\mathrm{sample}\;\left(\mathrm{substrate}\;\mathrm{and}\;\mathrm{biomass}\right)}\times 100$$

(5) $$\mathrm{Concentration}\;(\mathrm{mg}/L)\;=\frac{\mathrm{weight}\;\mathrm{of}\;\mathrm{GlcN}}{\mathrm{broth}\;\mathrm{volume}}$$

## 4. Conclusions

Solid-state fermentation to produce GlcN was performed herein. The maximal GlcN concentration was obtained when the fungi began to create a spore capsule, observed using a microscope. The moisture content in the solid substrate is important and should be larger than 80%. The initial supplemental aqueous solution volume was around 3 mL per gram of dry wheat bran. Supplemental mineral salts were necessary in this solid-state fermentation, with a concentration of around 0.5%.

## Figures and Tables

**Table 1:** The effect of water addition on the moisture content (MC) and water activity of wheat bran medium. a_w_: water activity.

Water Addition to the Medium (mL/g Wheat Bran)	MC in Wheat Bran (%, Wet Basis)	a_w_
Dry	0	0.401
0	0.112	0.572
0.3	0.317	0.887
0.6	0.445	0.930
0.9	0.532	0.966
1.2	0.596	0.992
1.8	0.683	0.999

## References

[1] Wen Z. Application and research progress of glucosamine. Proceedings of the International Symposium on the Frontiers of Biotechnology and Bioengineering.

[2] Arbia W., Arbia L., Adour L., Amrane A. (2013). Chitin extraction from crustacean shells using biological methods—A review. Food Technol. Biotechnol..

[3] Sitanggang A.B., Wu H.-S., Wang S.S., Ho Y.-C. (2010). Effect of pellet size and stimulating factor on the glucosamine production using *Aspergillus* sp. BCRC 31742. Bioresour. Technol..

[4] Nirmal N.P., Santivarangkna C., Rajput M.S., Benjakul S. (2020). Trends in shrimp processing waste utilization: An industrial prospective. Trends in Food Sci. Technol..

[5] Lopata A.L., Kleine-Tebbe J., Kamath S.D. (2016). Allergens and molecular diagnostics of shellfish allergy. Allergo J..

[6] Hsieh J.W., Wu H.S., Wei Y.H., Wang S.S. (2007). Determination and kinetics of producing glucosamine using fungi. Biotechnol. Prog..

[7] Sitanggang A.B., Sophia L., Wu H. (2012). MiniReview Aspects of glucosamine production using microorganisms. Int. Food Res. J..

[8] Habibi A., Karami S., Varmira K., Hadadi M. (2020). Key parameters optimization of chitosan production from *Aspergillus terreus* using apple waste extract as sole carbon source. Bioprocess Biosyst. Eng..

[9] Ruiz-Terán F., David Owens J. (1996). Chemical and enzymic changes during the fermentation of bacteria-free soya bean tempe. J. Sci. Food Agric..

[10] Margulies M., Egholm M., Altman W.E., Attiya S., Bader J.S., Bemben L.A., Berka J., Braverman M.S., Chen Y.-J., Chen Z. (2005). Genome sequencing in microfabricated high-density picolitre reactors. Nature.

[11] Wilhelm S.M., Carter C., Tang L., Wilkie D., McNabola A., Rong H., Chen C., Zhang X., Vincent P., McHugh M. (2004). BAY 43-9006 exhibits broad spectrum oral antitumor activity and targets the RAF/MEK/ERK pathway and receptor tyrosine kinases involved in tumor progression and angiogenesis. Cancer Res..

[12] Chang Y.F., Sitanggang A.B., Wu H.S. (2011). Optimizing biotechnological production of glucosamine as food ingredient from *Aspergillus* sp. BCRC 31742. J. Food Technol..

[13] Wu H.S., Lin B.C. (2017). Effect of oxygen transfer and pellet size for producing of glucosamine using *Aspergillus sydowii* BCRC 31742 cultivated in a fermenter. J. Food Process. Technol..

[14] Couto S.R., Sanromán M.Á. (2006). Application of solid-state fermentation to food industry—A review. J. Food Eng..

[15] Wu H.S., Peng J.W. (2020). Solid Medium for Producing Glucosamine and Its Application. U.S. Patent.

[16] Zheng Z., Shetty K. (1998). Solid-state production of beneficial fungi on apple processing wastes using glucosamine as the indicator of growth. J. Agric. Food Chem..

[17] Amanullah A., Christensen L.H., Hansen K., Nienow A.W., Thomas C.R. (2002). Dependence of morphology on agitation intensity in fed-batch cultures of Aspergillus oryzae and its implications for recombinant protein production. Biotechnol. Bioeng..

[18] Mohammadi M., Zamani A., Karimi K. (2013). Effect of phosphate on glucosamine production by ethanolic fungus *Mucor indicus*. Appl. Biochem. Biotechnol..

[19] Wu H.S., Chen J.H. (2019). Medium for Producing Glucosamine. U.S. Patent.

[20] Sitanggang A.B., Wu H.S., Wang S.S. (2009). Determination of fungal glucosamine using HPLC with 1-napthyl isothiocyanate derivatization and microwave heating. Biotechnol. Bioprocess Eng..

[21] Gervais P., Molin P. (2003). The role of water in solid-state fermentation. Biochem. Eng. J..

[22] Koop T., Luo B., Tsias A., Peter T. (2000). Water activity as the determinant for homogeneous ice nucleation in aqueous solutions. Nature.

[23] Labuza T.P. (1980). The effect of water activity on reaction kinetics of food deterioration. Food Technol.

[24] Zhao S.M. (2012). Determination of water activity in buckwheat flour by way of Conway dish diffusion method. J. Anhui Agri. Sci..

